# Acute and chronic effects of *Rhaponticum carthamoides* and *Rhodiola rosea* extracts supplementation coupled to resistance exercise on muscle protein synthesis and mechanical power in rats

**DOI:** 10.1186/s12970-020-00390-5

**Published:** 2020-11-16

**Authors:** Rémi Roumanille, Barbara Vernus, Thomas Brioche, Vincent Descossy, Christophe Tran Van Ba, Sarah Campredon, Antony G. Philippe, Pierre Delobel, Christelle Bertrand-Gaday, Angèle Chopard, Anne Bonnieu, Guillaume Py, Pascale Fança-Berthon

**Affiliations:** 1 DMEM, Université Montpellier, INRAE, INRA UMR 866 - 2 place Pierre Viala, Bat. 22, 34060 Montpellier, France; 2 Université de Nîmes, Laboratoire CHROME, Nîmes, France; 3 Naturex SA, Rue Pierre Bayle, 84911 Avignon Cedex 9, France

**Keywords:** *Rhodiola rosea*, *Rhaponticum carthamoides*, Resistance exercise, Protein synthesis, Muscle growth

## Abstract

**Background:**

Owing to its strength-building and adaptogenic properties, *Rhaponticum carthamoides* (*Rha*) has been commonly used by elite Soviet and Russian athletes. *Rhodiola rosea* (*Rho*) is known to reduce physical and mental fatigue and improve endurance performance. However, the association of these two nutritional supplements with resistance exercise performance has never been tested. Resistance exercise is still the best way to stimulate protein synthesis and induce chronic muscle adaptations. The aim of this study was to investigate the acute and chronic effects of resistance exercise coupled with *Rha* and *Rho* supplementation on protein synthesis, muscle phenotype, and physical performance.

**Methods:**

For the acute study, fifty-six rats were assigned to either a trained control group or one of the groups treated with specific doses of *Rha* and/or *Rho*. Each rats performed a single bout of climbing resistance exercise. The supplements were administered immediately after exercise by oral gavage. Protein synthesis was measured via puromycin incorporation. For the chronic study, forty rats were assigned to either the control group or one of the groups treated with doses adjusted from the acute study results. The rats were trained five times per week for 4 weeks with the same bout of climbing resistance exercise with additionals loads. *Rha + Rho* supplement was administered immediately after each training by oral gavage.

**Results:**

The findings of the acute study indicated that Rha and *Rha + Rho* supplementation after resistance exercise stimulated protein synthesis more than resistance exercise alone (*p* < 0.05). After 4 weeks of training, the mean power performance was increased in the *Rha + Rho* and *Rha*-alone groups (*p* < 0.05) without any significant supplementation effect on muscle weight or fiber cross-sectional area. A tendency towards an increase in type I/ type II fiber ratio was observed in *Rha/Rho*-treated groups compared to that in the trained control group.

**Conclusion:**

*Rhodiola* and *Rhaponticum* supplementation after resistance exercise could synergistically improve protein synthesis, muscle phenotype and physical performance.

**Supplementary Information:**

The online version contains supplementary material available at 10.1186/s12970-020-00390-5.

## Background

*Rhaponticum carthamoides (Willd.)*, commonly known as Maral root or Rhaponticum, is a perennial herb found in the Altai and Saian Mountains of South Siberia and has been introduced in various regions of Central and Eastern Europe in the last few decades [1]. Currently, *Rhaponticum* is used in preparations such as dietary supplements for its adaptogenic and tonic properties that promote muscle growth and increase the body’s resistance to stress, such as trauma and fatigue [2]. In the last century, the muscle- and strength-building qualities of *Rhaponticum* have been largely investigated in Russia, where various preparations were commonly used by elite Soviet and Russian athletes who were exhausted by hard training to increase psychological and physical performance [3]. Several different classes of compounds have been previously isolated from various parts of *Rhaponticum*, mainly steroids, particularly ecdysteroids, and phenolics [1]. Ecdysteroids affect certain major metabolic pathways in mammals: protein synthesis, lipid metabolism and carbohydrate metabolism [4]. A number of research studies, which are not currently available in English [5], suggest that phytoecdysteroids (PEs) possess a broad spectrum of biological, pharmacological, and medicinal properties in mammals without androgenic effects. 20-Hydroxyecdysone (20HE), also called ecdysone or ecdysterone, is one of the main ecdysteroids present in *Rhaponticum*, comprising 0.1 to 1% of the dry matter of roots [6].

*Rhodiola rosea* (golden root, roseroot) is a plant that grows in the mountainous and arctic regions of North America, Europe, and Asia. *Rhodiola* is known to reduce physical and mental fatigue, improve cognitive function, and exert antidiabetic effects. The functional claim of *Rhodiola* dietary supplements mentioned in the consolidated list of Article 13 Health Claims of the European Food Safety Authority (EFSA) is that it “contributes to optimal mental and cognitive activity”. *Rhodiola* contains a range of biologically active substances, including organic acids, flavonoids, tannins and high amounts of rosavins (rosin, rosavin, rosarian), which are *Rhodiola*-specific glycosides, and salidroside, which are present in all species of *Rhodiola* [7]. *Rhodiola* is used to improve cognitive function and endurance performance, reduce mental fatigue and reactive oxygen species (ROS) production, and exert antidiabetic effects [7]. Interestingly, it has been shown that *Rhodiola* exhibits antidepressant, adaptogenic, anxiolytic-like, and stimulating effects in mice [8]. The molecular mechanisms underlying the effects of *Rhodiola* are currently unknown, although it has been hypothesized that it enhances the activity of monoamines and opioid peptides [9]. Thus, *Rhodiola* could improve the consumption of substrates, enhancing lipid oxidation and sparing glycogen [7].

Given the current knowledge on the effects of both plant extracts, their use to promote human health in both preventive and curative applications appears justified. On one hand, *Rhaponticum*-based supplementation has been shown to increase body weight and muscle mass [2] On the other hand, some ergogenic effects of *Rhodiola* supplementation have been found but most of them derive from behavioral effects and not, for the moment, from effects on muscle physiology [8] As athletic performance involves both central (behavioral) and peripheral (muscular) qualities, it became evident that we should test their combination. Moreover, as some of their biological effects may improve muscle function, one can expect that supplementation with mixed extracts could increase muscle performance. Indeed, it is essential for athletes to ensure that they have optimal amounts of muscle mass for maximum performance.

Most animal studies that address muscle mass gain have focused their attention on PE, especially 20HE, but no study has investigated the effect of a combination of *Rhaponticum* and *Rhodiola*. Moreover, although positive effects of ecdysterone have been reported, significant data are not available, making evaluation of the experimental design and quality of the research difficult [10]; currently, it is hard to draw robust conclusions regarding the efficacy of supplements containing *Rhaponticum* in humans. The current study aimed to investigate the acute and chronic effects of resistance exercise and supplementation with *Rhaponticum* and *Rhodiola* on protein synthesis, muscle phenotype, and physical performance.

The primary endpoint of the chronic study was to identify the effects of *Rhaponticum* and *Rhodiola* and their potential synergistic effects on muscle mass and physical performance following resistance training. We designed an acute study to verify the potentially marked stimulation of muscle protein synthesis (MPS) by Rhaponticum in the decay of a single bout of resistance exercise and to examine whether adding Rhodiola plant extracts would affect Rhaponticum’s stimulatory effect.

## Methods

### Ethics and animal care

This study was approved by the Committee on the Ethics of Animal Experiments of Languedoc Roussillon in accordance with the guidelines of the French National Research Council for the Care and Use of Laboratory Animals (Permit Number APAFIS#713-201505261345689v3). Wistar Han rats were purchased from Charles River (Charles River Laboratories, L’Arbresle, Rhône, France). They were housed in pairs (Eurostandard type III H cage) at a constant room temperature (21 °C ± 1.5) and maintained in a 12/12 h light/dark cycle. Wood sticks (Top Brick rats, SAFE, Augy, France) were added for enrichment during husbandry and experimentation periods. Animals were acclimated for 1 week before experimental procedures with daily handling.

### Acute study design

#### Animals

Eleven-week-old Wistar Han rats (*n* = 56) were fed a specialized A04 low-protein 10% diet (30 g/day; protein, antioxidant, and vitamin content to mimic the self-administered, unfortified diet of humans) obtained from Scientific Animal Food & Engineering (SAFE, Augy, France), and water was given ad libitum. On day 1 of the experiment, the rats were 12 weeks old (315 +/− 10 g) and were considered adults.

Rats were randomly assigned to one of the 7 groups (*n* = 8 per group) defined by supplement treatment, as shown in Table 1. Animal doses were chosen based on human doses.

**Table 1:** Human equivalent doses (HEDs) administered to the different groups of animals in the acute study

Conversion of human dose to rat dose	Daily rat dose (mg/kg body weight)	HED (mg/kg)^**a**^	HED (mg/day)
Control group (CTL)	**–**	**–**	**–**
*Rhodiola* group (*Rho*)	43.5	8.33	500
*Rhaponticum* group (*Rha*)	43.5	8.33	500
*Rhaponticum/Rhodiola* 50–50 dose 1 group (*Rha + Rho* D1)	87.1	16.67	1000
*Rhaponticum/Rhodiola* 50–50 dose 2 group (*Rha + Rho* D2)	43.5	8.33	500
*Rhaponticum/Rhodiola* 50–50 dose 3 group (*Rha + Rho* D3)	21.8	4.17	250
*Rhaponticum/Rhodiola* 50–50 dose 4 group (*Rha + Rho* D4)	8.7	1.67	100

^a^ Formula from FDA, 2005; Human equivalent dose (HED; mg/kg) = animal dose in mg/kg x (animal weight in kg/human weight in kg)^033^

#### Resistance exercise and supplement administration

Rats were exercised on an apparatus adapted from a previous study [11]. A 1-m high ladder with 2-cm grid steps and 85° incline was made in our laboratory. First, the rats were familiarized with the ladder by voluntarily climbing it from the bottom to the top cage for 1 week without any additional load. During the experiment, cloth bags containing weights were attached to the base of the tail with a Velcro strap. After 1 week of familiarization, eight rats were randomly assigned to each of the seven groups as defined above. On the day of the experiment, food was withdrawn 4 h before the single bout of exercise. The rats in each group performed 10 climbs without an additional load and then performed 10 climbs carrying 50 and 75% of their body mass. Between each climb, the rats were allowed to rest for 2 min, and they were allowed to rest for 5 min between the two sets of 10 climbs. Immediately after the single bout of resistance exercise, the rats were put in their cages where the supplement was administered, and the rats were kept fasted until anesthesia (the rats were only provided free access to water).

*Rhaponticum carthamoides* and *Rhodiola rosea* L. extracts used in this study were kindly provided by Naturex (Avignon, France) and were prepared according to a patented method (US9700589B2, WO2016/125025). *Rhodiola* hydro-alcoholic extract was standardized to > 2% rosavins (rosin, rosarin and rosavin) and, also, contained a minimum of 1% salidroside. *Rhaponticum* hydro-alcoholic extract contained 20-hydroxyecdysone (20HE) or β-ecdysone (0.4%) and other phytoecdysteroids (total of 0.7%).

*Rhaponticum* and *Rhodiola* extracts and different combination of both (*Rha + Rho)* were administered immediately after exercise by oral gavage (2 ml). Solutions were extemporaneously prepared in 0.5% carboxymethyl cellulose (CMC).

#### Muscle sampling and protein extraction

Two hours after supplement gavage, the rats were intraperitoneally injected with 10 mM puromycin (100 μL of puromycin solution/25 g body weight, Sigma Aldrich, Saint-Louis, Missouri, USA). Twenty-five minutes after puromycin injection, animals were euthanized via an intraperitoneal injection of pentobarbital 150 mg.kg^− 1^ (Pentobarbital®, Ceva, Libourne, France). Thirty minutes after the puromycin injection, the right flexor digitorum profundus (FDP), deltoid and biceps muscles were harvested, frozen in nitrogen-cooled isopentane, and stored at − 80 °C until biochemical analysis. Twenty milligrams of each muscle were homogenized in 10 volumes of lysis buffer (20 mM Tris at pH 7.5, 150 mM NaCl, 2 mM EDTA, 1% Triton X-100) with protease inhibitor cocktails (P8340, Sigma Aldrich). The homogenate was centrifuged at 10,000×g for 10 min at 4 °C, and the supernatant was collected.

#### Protein synthesis measurement

Protein synthesis was measured by surface sensing of translation, as previously described [12]. Puromycin incorporation in proteins was assessed by immunoblotting on 4–20% acrylamide gels. Protein samples (50 μg) were denatured, separated by 10% SDS-PAGE, and transferred onto nitrocellulose membranes. An anti-puromycin primary antibody (anti-puromycin antibody (1/3000), clone 12D10 from EMD Millipore, Burlington, Massassuchets, USA) was applied overnight at 4 °C, and the membrane was subsequently incubated with secondary antibody (1/4000) conjugated to peroxidase (anti-mouse IgG; ECL from GE Healthcare UK Limited, Amersham, UK) The optical density of the entire sample lane was assessed and normalized with Ponceau S total protein staining.

### Chronic study design

#### Animals

Eleven-week-old Wistar Han rats (*n* = 40) were fed 30 g/day of the specialized low-protein food A04, previously designed and obtained from SAFE (Augy, France), and water was provided ad libitum. On day 1 of the experiment, the rats were 12 weeks old (334.4 ± 10 g) and were considered adults.

According to the results obtained during the acute phase of the study, we retained the *Rha + Rho* mix dose that produced the greater effect on protein synthesis, i.e. HED = 500 mg (50–50%). Four groups were defined and received treatment as shown in Table 2.

**Table 2:** Human equivalent doses (HEDs) used to feed the different groups of animals in the chronic study

Conversion of the human dose to rat dose	Daily rat dose (mg/kg BW)	HED (mg/kg BW)^**a**^	HED (mg/day)
Control group (CTL)	**–**	**–**	**–**
*Rhodiola* group (Rho)	21.8	4.17	250
*Rhaponticum* group (*Rha*)	21.8	4.17	250
*Rhaponticum/Rhodiola* 50–50 group (*Rha + Rho*)	43.5	8.33	500

^a^ Formula from FDA, 2005; Human equivalent dose (HED; mg/kg) = animal dose in mg/kg x (animal weight in kg/human weight in kg)^0.33^. *BW* body weight

#### Resistance training protocol and supplement administration

All rats of the four groups underwent a 4-week progressive resistance exercise program with additional loads. The exercise consisted of spontaneously climbing a 1-m-high ladder with 2-cm grid steps and inclined at 85°. Each training session consisted of one set of 20 repetitions with a 2 min rest between trials (5 min rest after the 10th trial, mid-exercise). The rats in the same cage were trained together. Training sessions were held 5 times per week, and the order of the groups was alternated. During the experiment, cloth bags containing weights were attached to the base of the tail with a Velcro strap. Three days before training, the rats were familiarized with the ladder by performing 3 climbs without additional load. On day 1 of the experiment, the additional load was 50% of the rat’s body mass, and the load was progressively increased to 150% of the rat’s body mass after 4 weeks. The supplements were immediately administered after each training session by oral gavage (2 ml). The solutions were extemporaneously prepared every morning. The vehicle was 0.5% CMC.

#### Training work and performance quantification

Training work (TW; in J) was calculated as the potential work developed during the training sessions using the following equation:

$$ \mathrm{TW}=\left(\mathrm{mload}+\mathrm{mrat}\right)\ast \mathrm{g}\ast \Delta \mathrm{h}\ast \mathrm{N} $$

where m is expressed in kg, g is the constant of the gravity on earth expressed in m.s^− 2^, Δh is the distance climbed in m, and N is the number of repetitions.

Performance was represented by the mechanical power output over the entire climbing session, calculated as the work performed against gravity (TW) divided by the total climbing time (s) and expressed in W:

$$ \mathrm{Performance}=\mathrm{TW}/\mathrm{time}. $$

The increase in power between the beginning and the end of the training program was calculated as the difference between the mean performance values on the 3 last days and the 3 first days of the training program. The full protocol has been previously described [11].

### One Repetition Maximum Test

On the last day of the experiment, the rats performed the one repetition maximum test (1-RM) to determine the maximal force a rat could produce after 19 training sessions; this value corresponds to the maximum load (additional load + body mass) that the animal could raise. The exercise consisted of climbing the ladder with progressively increasing loads (10% of BW increment), starting from 200% of body mass until failure, with a 5 min rest between each climb.

#### Muscle sampling

Between 72 h and 96 h after the end of the training program, muscles (right and left FDP, deltoid and biceps muscles) were harvested. The rats were euthanized by an intraperitoneal injection of pentobarbital (150 mg/kg). Muscles were weighed and immediately frozen in liquid nitrogen for biochemical studies. All samples were stored at − 80 °C.

#### Muscle cross-sectional area and fiber type distribution

Transverse serial sections of FDP muscle (10 μm thick) were obtained using a cryostat maintained at − 25 °C (HM-560, Microm H, Thermo Scientific, Waltham, Massassuchets, USA). Sections were stored at − 20 °C until histochemical staining. Before labeling, the sections were dried and fixed for 10 min in acetone. The sections were then washed in PBS, blocked and permeabilized with PBS 0.1% Triton X-100 and 20% horse serum.

For cross-sectional area (CSA) determination, the sections were incubated for 1 h with a rabbit anti-laminin antibody (1/400) (Sigma Aldrich, Saint-Louis, Missouri, USA), washed and then incubated with a secondary antibody conjugated to ALEXA 488 (goat anti-rabbit, Sigma, 1:800).

For muscle fiber typing determination, the sections were incubated with anti-MHC primary antibodies (anti-slow (I) MyHC, BA-D5, Developmental Studies Hybridoma Bank, 1:10 and anti-fast (II) MyHC, M4276, Sigma-Aldrich, 1:200) for 1 h at 37 °C, followed by washes in PBS and incubation with the secondary antibodies (1/800) (ALEXA 488; ALEXA 568, A11031, Invitrogen, Carlsbad, California, USA,) for 1 h.

The sections were scanned using a Nanozoomer (Hamamatsu), and CSA and fiber typing and were determined using ImageJ® software (version 1.46r).

#### Statistical analysis

All values are expressed as the mean ± SD. The normality of distributions was examined with the Shapiro-Wilk test. Since the studied variables displayed a normal distribution and a similar variance among groups, the effects were analyzed with a two-way ANOVA. In the case of a significant interaction effect, Fisher’s LSD post hoc tests were performed. Otherwise, the data were analyzed by the nonparametric Kruskal-Wallis and Dunn’s multiple comparisons tests. Analyses were performed on GraphPad Prism software (Prism 8, GraphPad software, La Jolla, CA, USA). The level of statistical significance was set at *p* < 0.05.

## Results

### Acute study

#### *Rhaponticum* coupled with *Rhodiola* stimulates MPS in active skeletal muscles

**Fig. 1:**
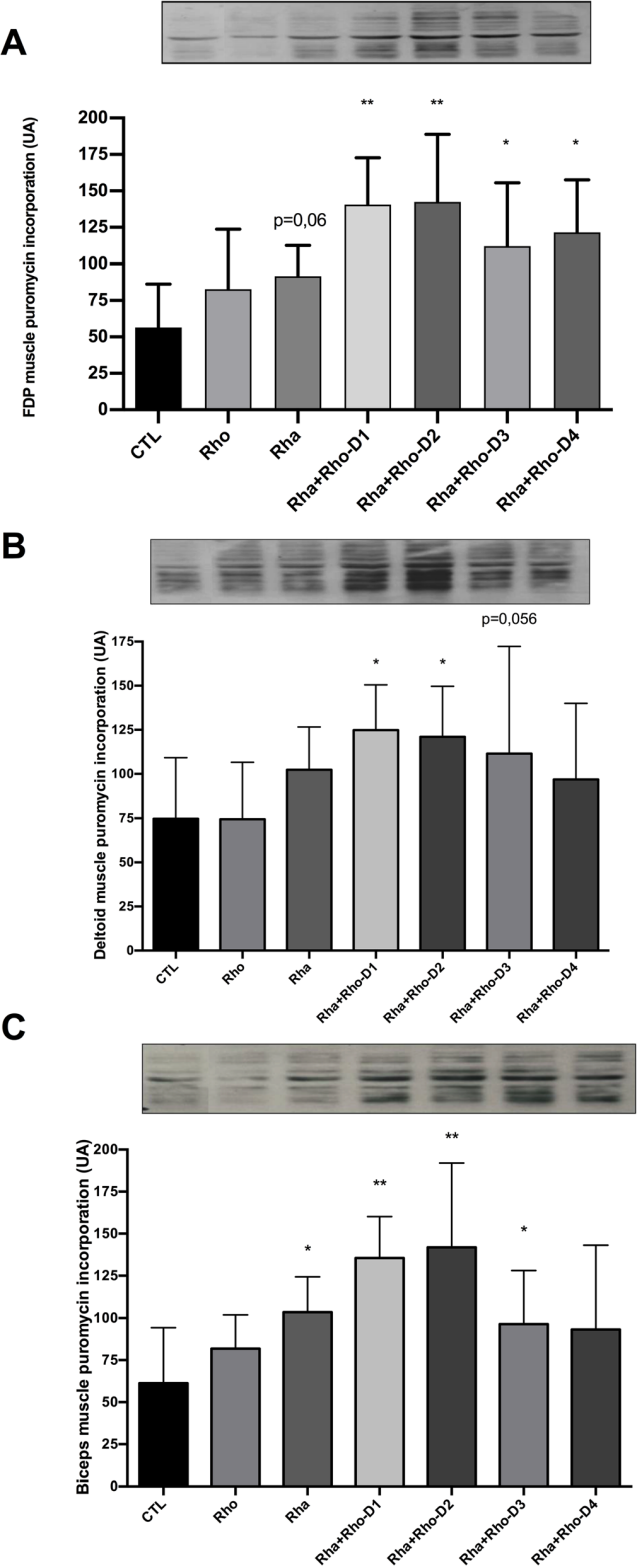
Effects of *Rhaponticum* and *Rhodiola* extracts on protein synthesis. Quantitative analysis of puromycin incorporation in total muscle protein extracts of FDP (**a**), deltoid (**b**) and biceps muscles (**c**) following acute resistance exercise. Graphs show the level of puromycin incorporation compared to the control (CTL) group. *, p < 0.05; * *, p < 0.005

Protein synthesis was upregulated in all *Rha + Rho*-treated groups compared to that in the control group (Fig. 1), whereas the rate of protein synthesis tended to increase with Rhaponticum treatment (*p* < 0.05 for biceps (Fig. 1c); *p* = 0.059 for FDP (Fig. 1a)). Thus, it appears that the mixture of *Rhaponticum* and *Rhodiola* plant extracts can stimulate skeletal MPS more efficiently than the administration of *Rhodiola* or *Rhaponticum* alone. The 500 mg dose of the 50–50% *Rha + Rho* mixture (dose 2) showed the greatest stimulation of MPS and we retained this dose for the chronic study.

Moreover, the results of the acute study clearly showed a synergistic effect of *Rhaponticum* plus *Rhodiola* for doses ranging from 250 to 1000 mg in a 50%/50% ratio. Among the two plant extracts, it also appeared that *Rhaponticum* could exert a major effect since the *Rhaponticum*-alone groups showed an increase, a strong tendency, and no effect in biceps, FDP, and deltoid muscles, respectively, whereas the *Rhodiola*-alone groups did not show any significant variation in muscle protein synthesis (Fig. 1).

### Chronic study

#### Body weight gain

**Fig. 2:**
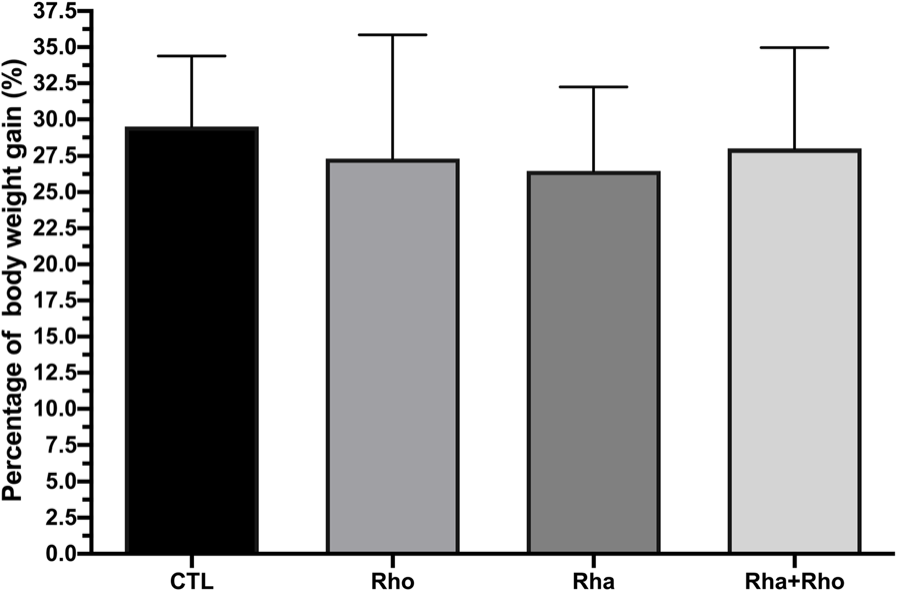
Effects of chronic *Rhaponticum* and *Rhodiola* extract treatments associated with exercise on body weight. Total mass was assessed before and after the chronic protocol

We assessed body weight before and after the training protocol, and surprisingly, we did not find a significant difference between the three supplementation groups and the trained control group: the body weight of each of these groups increased by 30% (Fig. 2).

#### Muscle mass gain

**Fig. 3:**
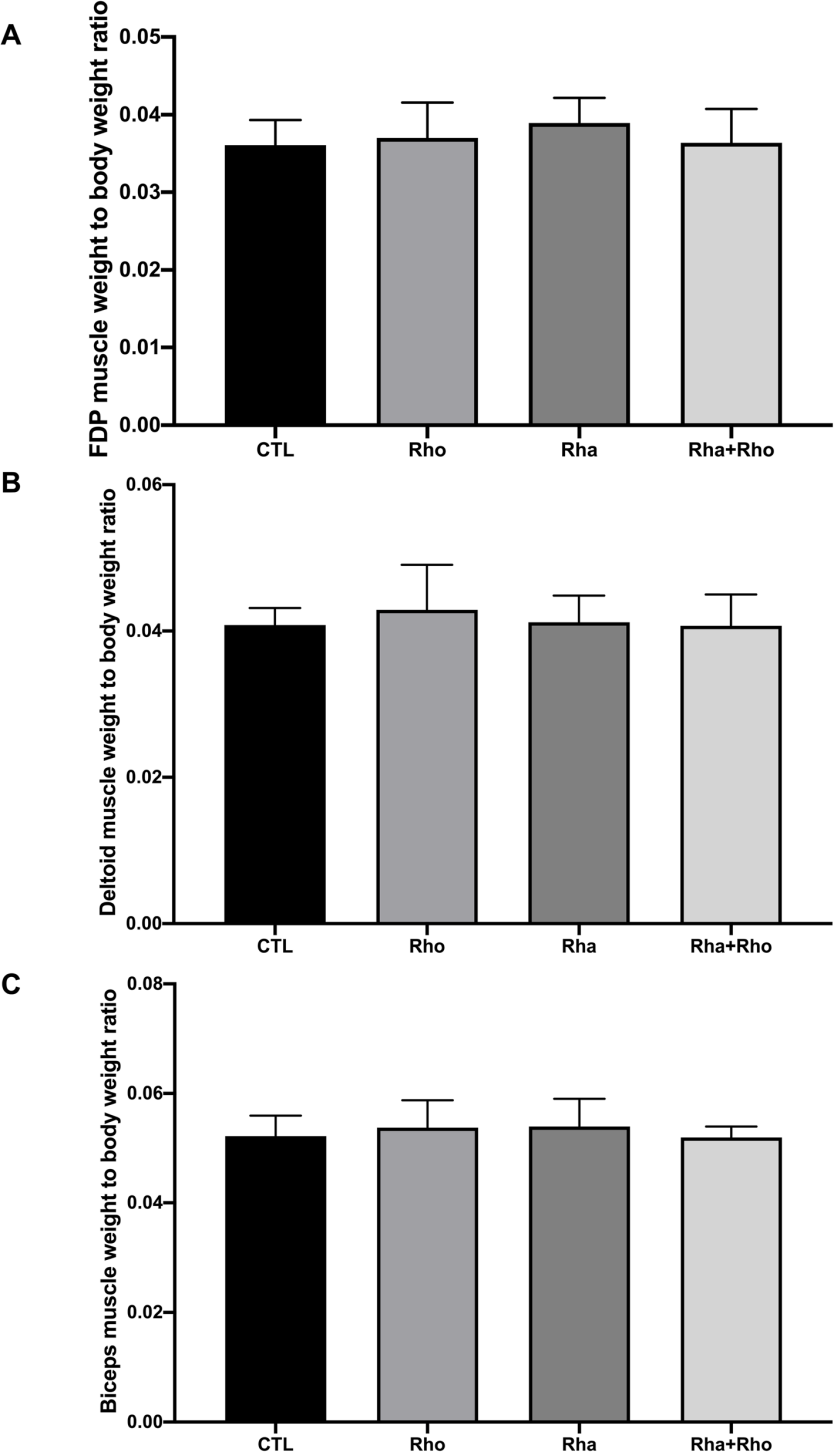
Effects of chronic *Rhaponticum* and *Rhodiola* extract treatments associated with exercise on muscle mass. Masses of FDP (**a**), deltoid (**b**) and biceps muscles (**c**) are normalized to total body weight. *: p < 0.05 compared to the CTL group

Since the acute study showed an increase in protein synthesis in the groups treated with *Rha + Rho,* we expected to observe an increase in muscle mass in animals subjected to resistance training, a condition known to generate muscle hypertrophy, in the chronic study. Fig. 3a, b and c show the relative muscle mass (i.e.*,* ratio of muscle mass/body mass) of each group. We did not find differences in the deltoid and biceps muscles between the treatment groups (*Rhodiola* and *Rhaponticum*) and the trained control group.

#### *Rhaponticum,* coupled with and without *Rhodiola,* increases mean power output following resistance training, but not one maximal repetition

**Fig. 4:**
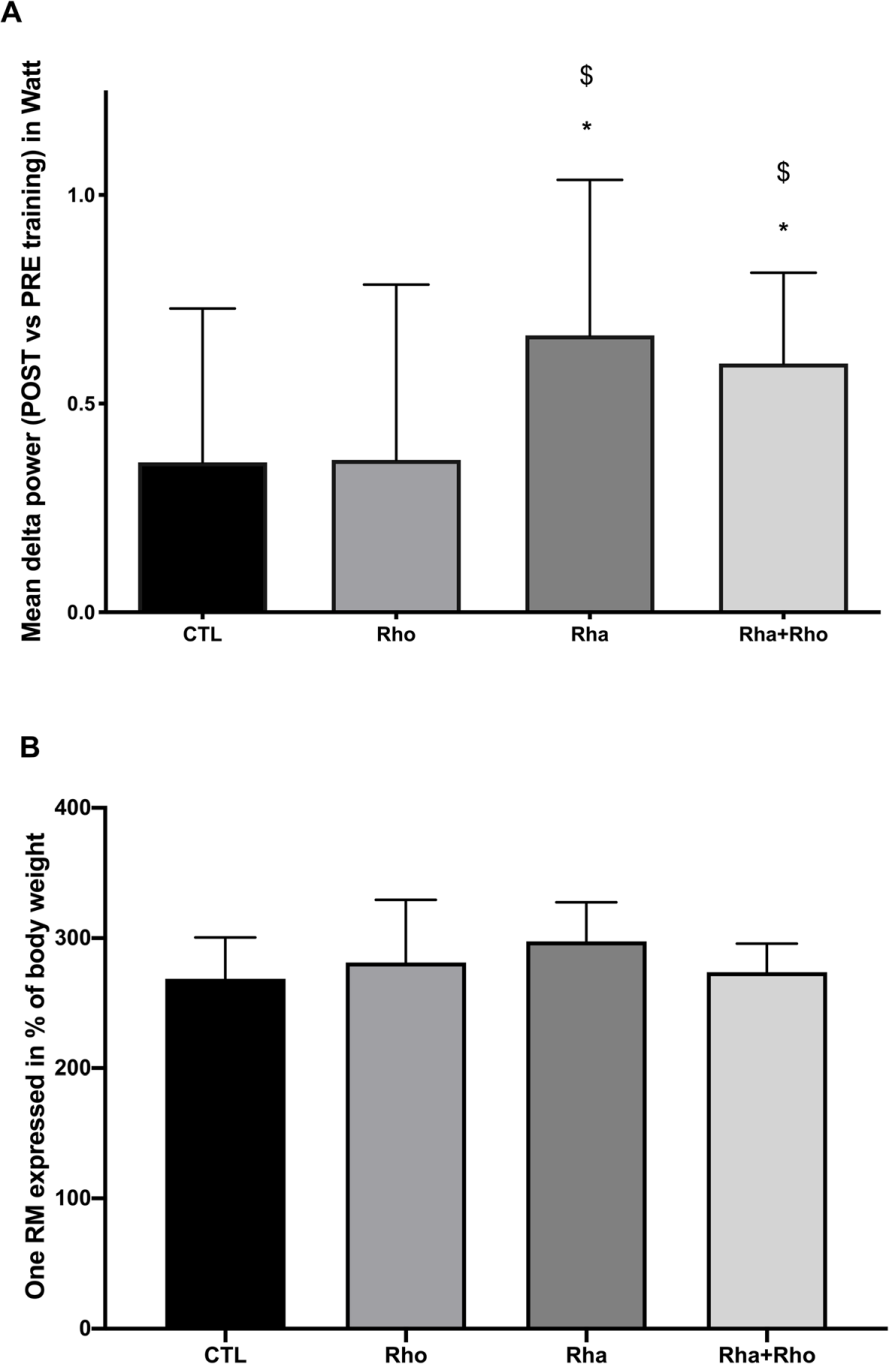
Effects of chronic *Rhaponticum* and *Rhodiola* extract treatments associated with exercise on physical performance. The graphs show the mean delta power between pre-training and post-training in each treatment group (**a**)*,* and for the 1-RM test (**b**). *: p < 0.05 compared to the CTL group. $: p < 0.05 compared to the Rho group

We noted a large interindividual variability in the training response between animals. Remarkable was the docile behavior of rats from the *Rho* group. Concerning the developed mechanical power measured in the climbing task, animals from both the *Rha* and *Rha + Rho* group showed an increase in developed mechanical power output after the training protocol compared to the CTL and *Rho* groups. There was no statistical difference between *Rha* and *Rha + Rho* groups. (Fig. 4a). In addition to the power performance test, animals were tested against their absolute strength gain with the one maximal repetition (1RM) test. Curiously, we did not find any difference in absolute strength gain with 1RM test after the resistance training protocol among the different groups (Fig. 4b).

#### *Rhaponticum* coupled with *Rhodiola* does not affect the cross-sectional area but tends to enhance the ratio of type I to type II muscle fiber in the flexor digitorum profundus

**Fig. 5:**
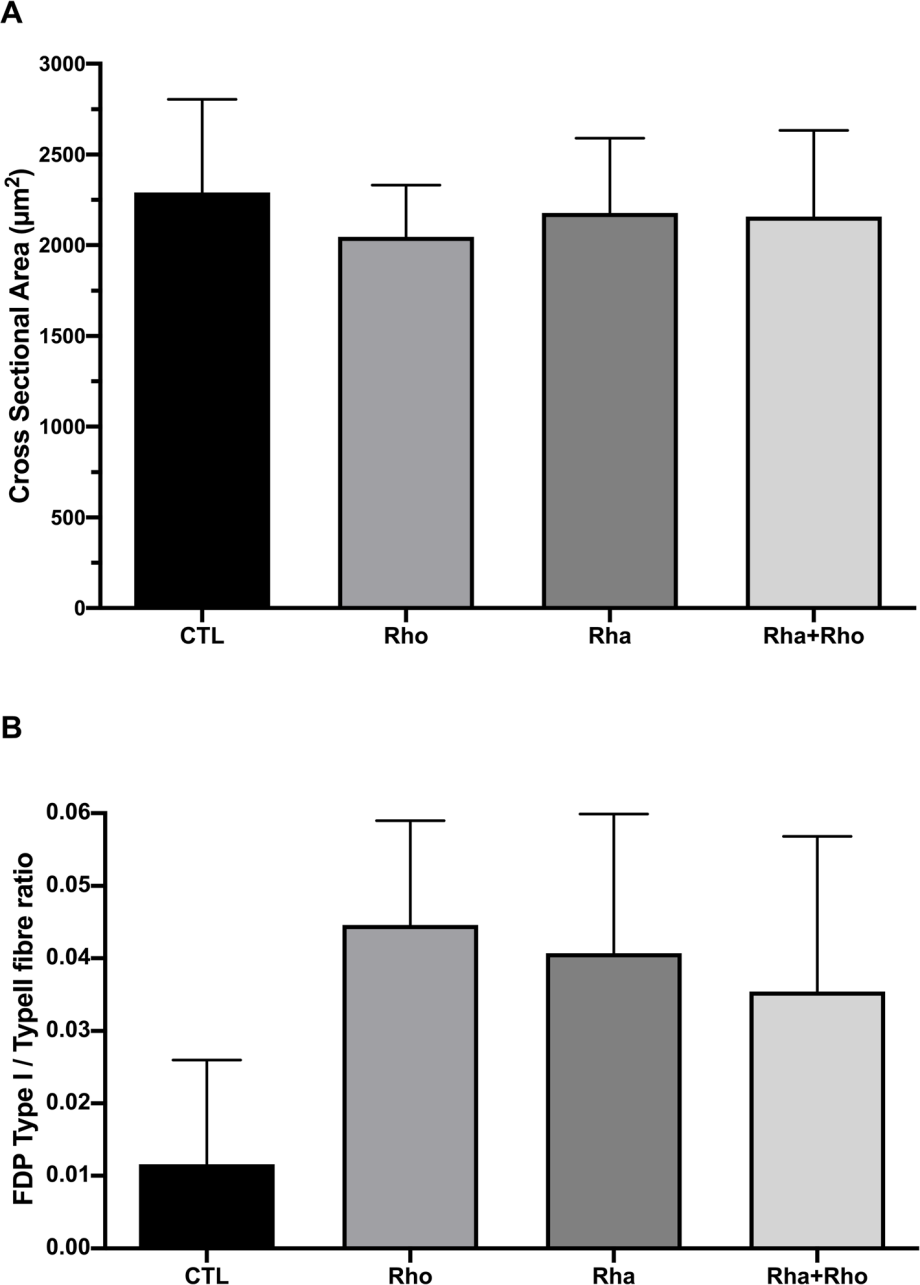
Effects of chronic *Rhaponticum* and *Rhodiola* extract treatments associated with exercise on muscle fibers (FDP muscle). The mean cross-sectional area of FDP muscle is presented in (**a**). The ratio of type I/type II fibers was evaluated (**b**)

Because FDP is the muscle most involved in climbing performance, we focused our attention on only this muscle [13]. Surprisingly, the results did not reveal any treatment effect on mean CSA (Fig. 5a). We measured the ratio of type I/type II fibers and found that this ratio tended to increase in the four supplementation groups (*p* = 0.09), compared with that in the trained control group (Fig. 5b).

## Discussion

This study was conducted to examine whether 1) supplementation of *Rhaponticum* could stimulate MPS and maintain this effect when combined with *Rhodiola*, and 2) chronic supplementation of *Rha + Rho* coupled with resistance training could also enhance physical performance. The acute study revealed that *Rhaponticum* could stimulate protein synthesis above placebo in response to resistance exercise. Interestingly, combining *Rhodiola* and *Rhaponticum* plant extracts stimulated higher muscle protein synthesis than *Rhaponticum* alone. When given chronically, *Rhaponticum* augmented mechanical power above placebo, but the combination of plant extracts did not further augment muscle performance above *Rhaponticum* plant extracts alone.

From a physiological point of view, physical performance is highly correlated with muscle mass which rely mostly upon protein synthesis in healthy conditions but also with motivational aspects. The rationale of the study was that chronic administration with a combination of Rhaponticum and Rhodiola plant extracts could enhance physical performance, taking advantage of the peripheral effect (protein synthesis) of Rhaponticum and adaptogenic effect of Rhodiola. We first designed an “acute” study in which we verified the potential stimulatory effect *Rha + Rho* supplementation on MPS in the context of a single bout of resistance exercise. Our first hypothesis was that *Rhaponticum* exerts an effect through enhanced muscle protein synthesis and that adding Rhodiola plant extracts does not affect Rhaponticum’s stimulatory effect.

We used a rat model of resistance exercise known to increase MPS [14]. The three main forearm muscles involved in the climbing activity were harvested (the FDP, deltoid, and biceps muscle) 2 hours after administration of supplements by oral gavage immediately after the exercise bout to ensure the animals were in the “anabolic window”. Interestingly, *Rhodiola* alone was not able to stimulate protein synthesis regardless of the muscle studied, while, *Rhaponticum* alone modestly increased protein synthesis in the biceps and FDP (*p* < 0.05 for biceps; *p* = 0.059 for FDP). However, when combined, *Rhaponticum* and *Rhodiola* plant extracts were able to increase protein synthesis at all dosages tested, except for the lowest dose (*Rha + Rho* D4). Subtle nuances were observed between the different active muscles, which were most likely directly linked to their implication in climbing movement and/or their phenotype. For example, the FDP muscle, which is the muscle most involved in this exercise model, responded to every *Rha + Rho* dose (from HED = 100 mg to 1000 mg) compared to the control group. Overall, the *Rha + Rho* doses that engendered the largest increase in protein synthesis after exercise were the three highest doses, i.e., HED = 1000, 500 and 250 mg, regardless of the muscle studied. Taken together, these results suggest that bioactive compounds in *Rhaponticum* extract were able to stimulate MPS and that some bioactive compounds in *Rhodiola* extract could exert a synergistic effect, as observed by puromycin incorporation (Fig. 1a, b and c).

This increase in protein synthesis induced by *Rha + Rho* could be explained at least in part by the anabolic effect of phytoecdysteroids, which are enriched in the extracts. Ecdysteroids are a class of polyhydroxylated ketosteroids with long carbon side-chains that are produced primarily in insects, and their analogues in plants are phytoecdysteroids such as *Rhaponticum*. Indeed, phytoecdysteroids have been shown to stimulate growth in several animal species, including mice [4, 15], rats, sheep, pigs, and quail [16]. The increased physical performance without training observed in a forced swim rat model is of particular interest [10]. In that study, in addition to increased performance, the authors found an increase in myofibrillar protein synthesis in the soleus and EDL muscles [10]. One of the most common ecdysteroids found in plants is 20HE. 20HE does not bind to the androgen receptor, suggesting that phytoecdysteroids, including 20HE, may exert their anabolic effect through an androgen-independent mechanism [17]. Some evidence indicates that ecdysteroids and 20HE activate Akt [18]. We performed western blotting of phosphorylated and total forms of Akt and we did not find any increase in the p-Akt/Akt ratio between the control and Rhaponticum or Rhaponticum+Rhodiola groups. Moreover, none of the downstream effectors i.e. mTOR, 4-EBP1 and rpS6, were upregulated (phosphorylated/total isoform ratio, see Additional figure 1). To date we have no explanation for the discrepancy between the acute increased protein synthesis and the absence of any significant stimulation of the mTOR pathway. Taken together, the results of the acute study confirmed that Rhaponticum alone is able to stimulate higher muscle protein synthesis than resistance exercise alone and even higher when combined with Rhodiola plant extracts, suggesting a synergistic effect. Further experiments investigating the role of the Akt/mTOR pathway in chronic supplementation are needed.

We next extended the study to chronic supplementation using, in addition to Rho or Rha doses alone (HED = 250 mg), the dose that best stimulated protein synthesis i.e. 500 mg *Rha + Rho* (dose 2) coupled with a 4-week resistance training program. In the chronic study, physical performance was evaluated using the mean mechanical power produced at the same relative workload before and after the training period. Compared to the control and *Rhodiola* groups, the *Rhaponticum* and *Rha + Rho* groups showed increases in climbing mechanical power output, suggesting that the repeated increases in muscle protein synthesis after each bout of resistance exercise could participate in physical performance. Surprisingly, if *Rhaponticum* augmented mechanical power above placebo, the combination of plant extracts did not further augment muscle performance above *Rhaponticum* plant extracts alone. Interestingly, compared to the trained control condition, *Rhodiola* alone did not elicit any increase in mechanical power (Fig. 4a). It was expected a synergistic effect of *Rhodiola*, due to its adaptogenic properties leading to an improved cognitive function [19] or at least in part coming from a reduced mental fatigue as described by [20] in young militaries. However, our results are in accordance with those of De Bock et al. (2004) [21], since they did not observe changes in muscle strength after 4 weeks of Rhodiola supplementation, as we didn’t notice any synergestic effect in chronic study. The mechanisms underlying the increased physical performance of all the groups include, but are not restricted to, the following: 1) an increase in motor command with an increase in the activated motor neuron pool and probably an increase in motor unit coordination, 2) an increase in fiber size and strength, 3) an increase in the power produced by each fiber type, and 4) an increase in myofibrillar ATPase activity for a given myosin isoform. Unfortunately, we did not measure the activation level; however, the hypothesis that *Rhaponticum* and/or *Rhodiola* extracts alters cortical output by modulating the motivation of the animals, leading to an increased physical performance, should not be excluded. In support, a stimulatory effect of intraduodenally administered doses of *Rhaponticum* extracts on the central nervous system of rats had been demonstrated [2]. Similarly, 20HE could produce an increase in acetylcholinesterase (AChE) in the rat brain [22], which could enhance learning capacity. Concerning the increase in fiber size and strength, our study lacks a true control (untrained) group to clearly show the effect of resistance training on fiber size. However, in a previous study that used rats of the same age and weight, we showed that the non-exercised control group had a mean FDP CSA value of 1440 ± 84 μm^2^ [14], which is notably lower than the value of 2291 ± 182 μm^2^ of the present trained control group. Clearly, the increase in strength is directly related to fiber CSA. It has been shown that resistance training can elicit a direct effect on developed power by each specific fiber type [23]. Indeed, resistance training can increase contraction velocity and absolute strength measured at the fiber level. Thus, even though we only observed a tendency of increase in the ratio of type I to type II fibers in the treated groups, these results do not exclude the possibility that the power of both fiber types was increased in response to the resistance training protocol. Indeed, after 4 weeks of resistance training using the same model, a mean increase in myofibrillar ATPase activity of 135.6% has been observed in FDP, biceps, and deltoid muscles, for a given myosin heavy chain isoform [24]. This effect could explain the increased performance of each treated group, except for the Rho group (Fig. 4b). In our experiments, *Rhodiola* was administered owing to its adaptogenic activity under strenuous physical effort, delaying fatigue and exerting metabolic effects such as promoting fatty acid utilization [7].

In order to a subsequent transfer to the clinic, requiring the product’s cost reduction especially for Rhodiola plant extracts, and based on the results of the acute study, we applied a combination consisted of the smallest 50%/50% dose of Rhaponticum plant extracts that had an effect on every muscle and the smallest 50%/50% dose of Rhodiola that had an effect at least in one studied muscle. Thus, we designed a mix dose of 175 mg *Rha* (70%) + Rho (30%) that was administered to animals that received also resistance training for 4 weeks. Interestingly, this combination dose produced the same enhancement in mechanical power as the 500 mg *Rha + Rho* 50%/50% dose (see Additional figure 4A). Further studies testing other *Rha + Rho* ratios and doses close to those used in this study should be performed to optimize the *Rha/Rho* ratio.

## Conclusion

When acutely administered and coupled with resistance exercise, *Rhaponticum* extract can increase MPS, and a synergistic effect could be expected when combined with *Rhodiola* extract. With chronic administration and resistance training, the same combination of *Rha + Rho* improves muscle power and strength performance without altering muscle mass and fiber CSA. Interestingly, another ratio (*Rha + Rho* 70–30%) had a significant effect on muscle mass in the chronic study suggesting that an optimized ratio of a given dose exists and further studies are scheduled in this sense. Aside these results, it is important to note that, although not evaluated in this study, *Rhodiola* and *Rhaponticum* alone, especially at high doses, may have opposite behavioral effects that may impact whole body performance. Currently, the underlying mechanisms leading to the increased MPS are still far from being unraveled. Further cellular and animal studies should address this question.

## Supplementary Information

**Additional file 1: Figure 1.** Effects of *Rhaponticum* and *Rhodiola* extracts on protein synthesis markers. Quantitative analysis of p-Akt/total Akt (1.A), p-mTOR/total mTOR (1.B), p-rpS6/total rpS6 (1.C) and p-4EBP-1/total 4EBP-1 (1.D) in total protein extracts of FDP muscle.

**Additional file 2: Figure 2.** Effects of chronic *Rhaponticum* and *Rhodiola* extract treatments (at the dose of 175 mg *Rha* (70%) + Rho (30%)) associated with exercise on body weight. Total mass was assessed before and after the chronic protocol.

**Additional file 3: Figure 3.** Effects of chronic *Rhaponticum* and *Rhodiola* extract treatments (at the dose of 175 mg *Rha* (70%) + Rho (30%)) associated with exercise on muscle mass. Masses of FDP (A), deltoid (B) and biceps muscle (C) are normalized to total body weight. *: p < 0.05 compared to the CTL group (TIFF 1428 kb)

**Additional file 4: Figure 4.** Effects of chronic *Rhaponticum* and *Rhodiola* extract treatments (at the dose of 175 mg *Rha* (70%) + Rho (30%)) associated with exercise on physical performance. The graphs show the mean delta power between pre-training and post-training in each treatment group (4.A)*,* and for the 1-RM test (4.B). *: p < 0.05 compared to the CTL group. $: p < 0.05 compared to the Rho group. (TIFF 1135 kb)

**Additional file 5: Figure 5.** Effects of chronic *Rhaponticum* and *Rhodiola* extract treatments (at the dose of 175 mg *Rha* (70%) + Rho (30%)) associated with exercise on muscle fibers (FDP muscle). The mean cross-sectional area of FDP muscle is presented in Fig. (5.A). The ratio of type I/type II fibers was evaluated (5.B).

**Additional file 6: Figure 6. **Illustrations of Western Blot quantitative analysis of p-Akt/total Akt, p-mTOR/total mTOR, p-rpS6/total rpS6 and p-4EBP-1/total 4EBP-1 in total protein extracts of FDP muscle, with Stainfree as internal control.

**Additional file 7: Figure 7. **Illustrations of Ponceau as internal control for analysis of puromycin incorporation in total muscle protein extracts of FDP (A), deltoid (B) and biceps muscles (C). 

**Additional file 8: **Additional methods.

## Data Availability

The datasets generated and/or analyzed during the current study are not publicly available due to intellectual property rights but are available from the corresponding author on reasonable request.

## References

[1] Kokoska L, Janovska D (2009). Chemistry and pharmacology of Rhaponticum carthamoides: a review. Phytochemistry.

[2] Petkov V, Roussinov K, Todorov S, Lazarova M, Yonkov D, Draganova S (1984). Pharmacological investigations on Rhaponticum carthamoides. Planta Med.

[3] Azizov AP, Seĭfulla RD, Ankudinova IA, Kondrat’eva II, Borisova IG (1998). The effect of the antioxidants Elton and leveton on the physical work capacity of athletes. Eksp Klin Farmakol.

[4] Tóth N, Szabó A, Kacsala P, Héger J, Zádor E (2008). 20-Hydroxyecdysone increases fiber size in a muscle-specific fashion in rat. Phytomedicine Int J Phytother Phytopharm.

[5] Syrov VN, Kurmukov AG (1976). Anabolic activity of phytoecdysone-ecdysterone isolated from Rhaponticum carthamoides (Willd.) Iljin. Farmakol Toksikol.

[6] Shanely KAZAR, Merritt EK, Mcbride JM (2013). Phytoecdysteroids: a novel, non-androgenic alternative for muscle health and performance.

[7] Huang S-C, Lee F-T, Kuo T-Y, Yang J-H, Chien C-T (2009). Attenuation of long-term Rhodiola rosea supplementation on exhaustive swimming-evoked oxidative stress in the rat. Chin J Physiol.

[8] Mattioli L, Funari C, Perfumi M (2009). Effects of Rhodiola rosea L. extract on behavioural and physiological alterations induced by chronic mild stress in female rats. J Psychopharmacol Oxf Engl.

[9] Kelly GS (2001). Rhodiola rosea: a possible plant adaptogen. Altern Med Rev J Clin Ther.

[10] Chermnykh NS, Shimanovskiĭ NL, Shutko GV, Syrov VN (1988). The action of methandrostenolone and ecdysterone on the physical endurance of animals and on protein metabolism in the skeletal muscles. Farmakol Toksikol.

[11] Philippe AG, Py G, Favier FB, Sanchez AMJ, Bonnieu A, Busso T (2015). Modeling the responses to resistance training in an animal experiment study. Biomed Res Int.

[12] Goodman CA, Hornberger TA (2013). Measuring protein synthesis with SUnSET: a valid alternative to traditional techniques?. Exerc Sport Sci Rev.

[13] MacLeod D, Sutherland DL, Buntin L, Whitaker A, Aitchison T, Watt I (2007). Physiological determinants of climbing-specific finger endurance and sport rock climbing performance. J Sports Sci.

[14] Begue G, Douillard A, Galbes O, Rossano B, Vernus B, Candau R (2013). Early activation of rat skeletal muscle IL-6/STAT1/STAT3 dependent gene expression in resistance exercise linked to hypertrophy. PLoS One.

[15] Todorov IN, Mitrokhin YI, Efremova OI, Sidorenko LI (2000). The Effect of Ecdysterone on the Biosynthesis of Proteins and Nucleic Acids in Mice. Pharm Chem J..

[16] Lafont R, Dinan L (2003). Practical uses for ecdysteroids in mammals including humans: an update. J Insect Sci Online.

[17] Gorelick-Feldman J, MacLean D, Ilic N, Poulev A, Lila MA, Cheng D (2008). Phytoecdysteroids increase protein synthesis in skeletal muscle cells. J Agric Food Chem.

[18] Gorelick-Feldman J, Cohick W, Raskin I (2010). Ecdysteroids elicit a rapid Ca2+ flux leading to Akt activation and increased protein synthesis in skeletal muscle cells. Steroids.

[19] Walker TB, Robergs RA (2006). Does Rhodiola rosea possess ergogenic properties?. Int J Sport Nutr Exerc Metab.

[20] Shevtsov VA, Zholus BI, Shervarly VI, Vol’skij VB, Korovin YP, Khristich MP (2003). A randomized trial of two different doses of a SHR-5 Rhodiola rosea extract versus placebo and control of capacity for mental work. Phytomedicine Int J Phytother Phytopharm.

[21] De Bock K, Eijnde BO, Ramaekers M, Hespel P (2004). Acute Rhodiola rosea intake can improve endurance exercise performance. Int J Sport Nutr Exerc Metab.

[22] Catalan RE, Aragones MD, Godoy JE, Martinez AM (1984). Ecdysterone induces acetylcholinesterase in mammalian brain. Comp Biochem Physiol C.

[23] Malisoux L, Francaux M, Nielens H, Theisen D (2006). Stretch-shortening cycle exercises: an effective training paradigm to enhance power output of human single muscle fibers. J Appl Physiol Bethesda Md 1985.

[24] Philippe AG, Lionne C, Sanchez AMJ, Pagano AF, Candau R (2019). Increase in muscle power is associated with myofibrillar ATPase adaptations during resistance training. Exp Physiol.

