# Supplementary figures and images for: Acute and chronic effects of Rhaponticum carthamoides and Rhodiola rosea extracts supplementation coupled to resistance exercise on muscle protein synthesis and mechanical power in rats
Source: J Int Soc Sports Nutr. 2020 Nov 16;17:58. doi: 10.1186/s12970-020-00390-5 (PMC7670727; doi:10.1186/s12970-020-00390-5)

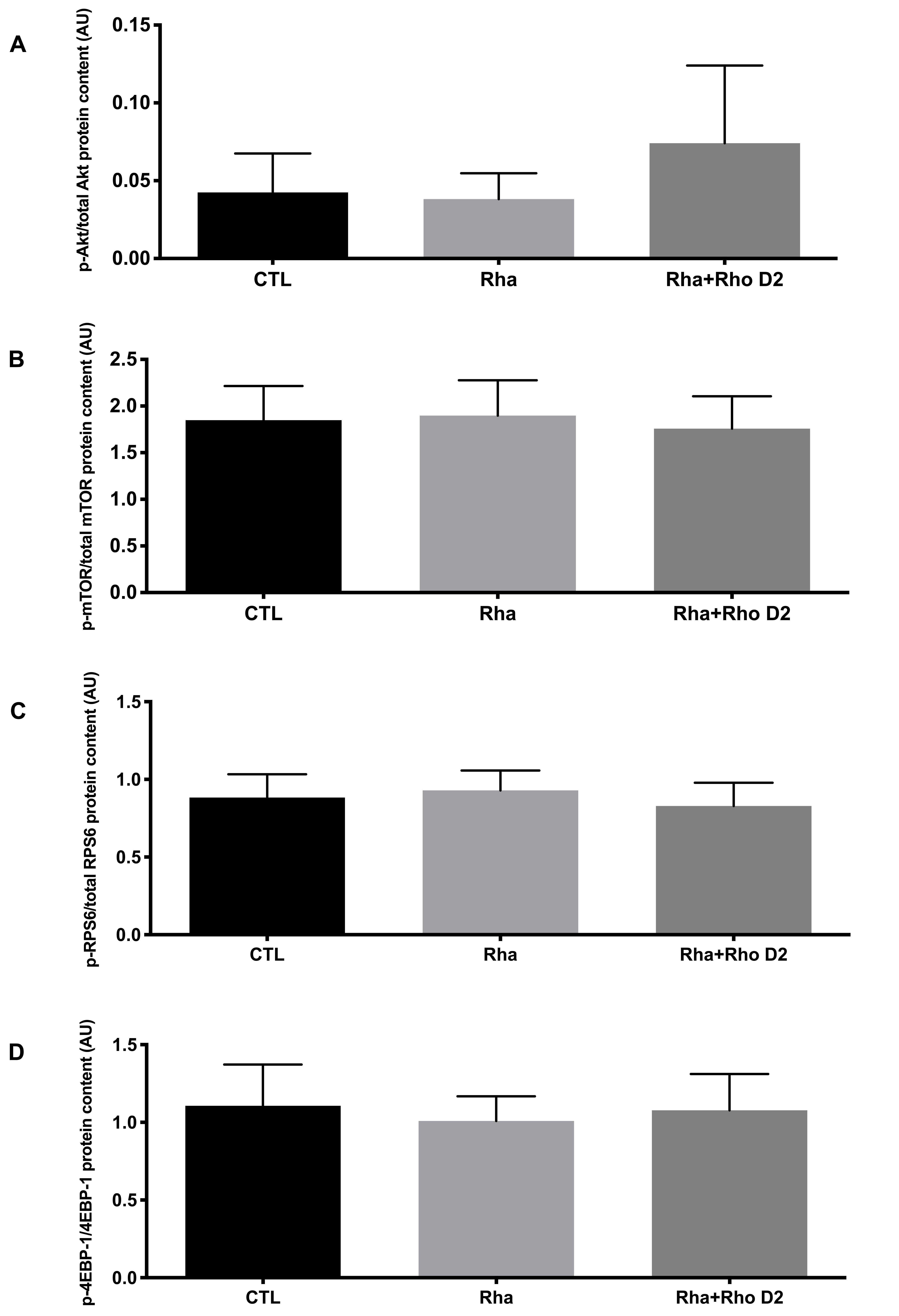

Supplement: Supplementary file 1 — Additional file 1: Figure 1. Effects of Rhaponticum and Rhodiola extracts on protein synthesis markers. Quantitative analysis of p-Akt/total Akt (1.A), p-mTOR/total mTOR (1.B), p-rpS6/total rpS6 (1.C) and p-4EBP-1/total 4EBP-1 (1.D) in total protein extracts of FDP muscle. [file 12970_2020_390_MOESM1_ESM.tiff]

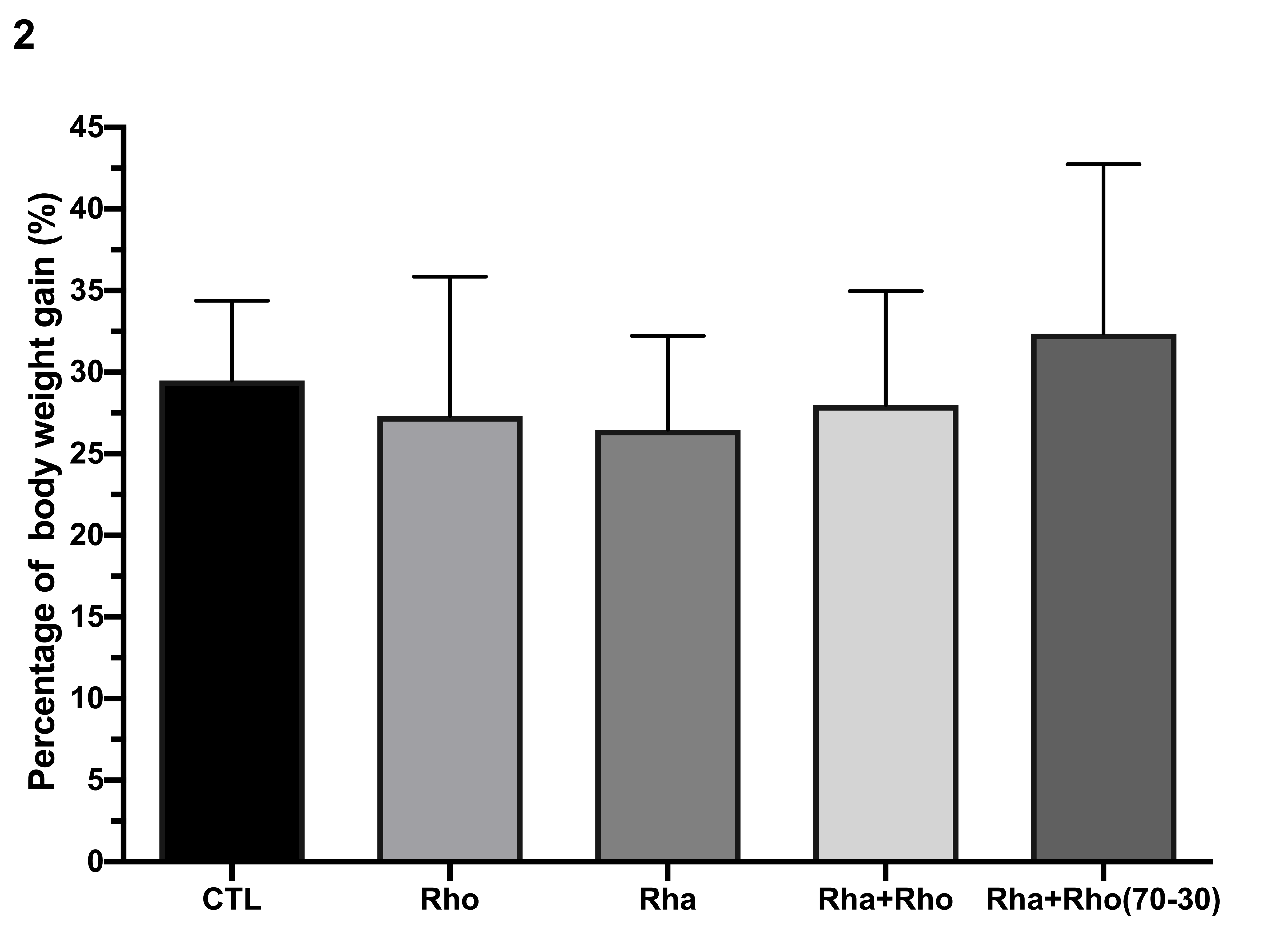

Supplement: Supplementary file 2 — Additional file 2: Figure 2. Effects of chronic Rhaponticum and Rhodiola extract treatments (at the dose of 175 mg Rha (70%) + Rho (30%)) associated with exercise on body weight. Total mass was assessed before and after the chronic protocol. [file 12970_2020_390_MOESM2_ESM.tiff]

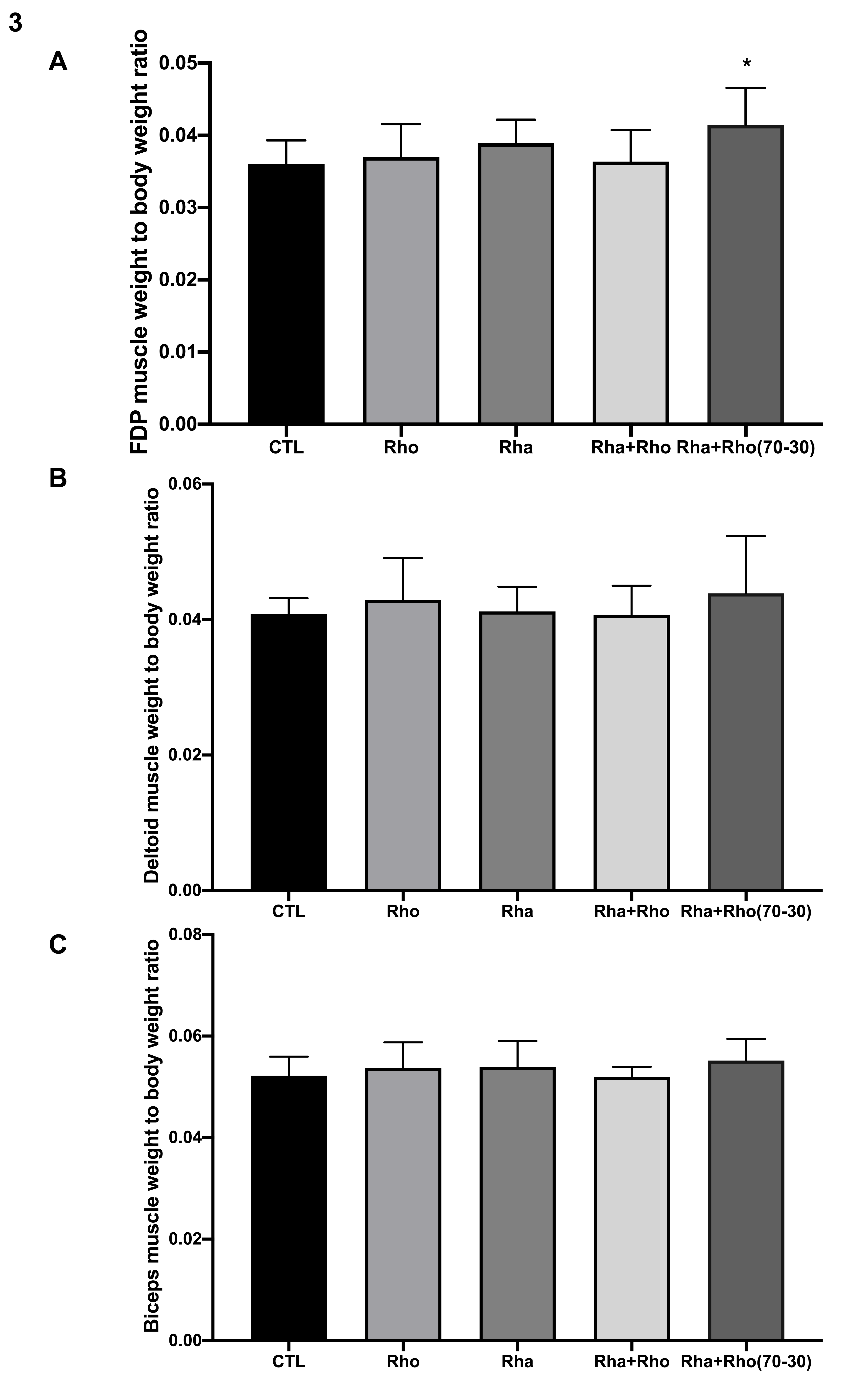

Supplement: Supplementary file 3 — Additional file 3: Figure 3. Effects of chronic Rhaponticum and Rhodiola extract treatments (at the dose of 175 mg Rha (70%) + Rho (30%)) associated with exercise on muscle mass. Masses of FDP (A), deltoid (B) and biceps muscle (C) are normalized to total body weight. *: p < 0.05 compared to the CTL group (TIFF 1428 kb) [file 12970_2020_390_MOESM3_ESM.tiff]

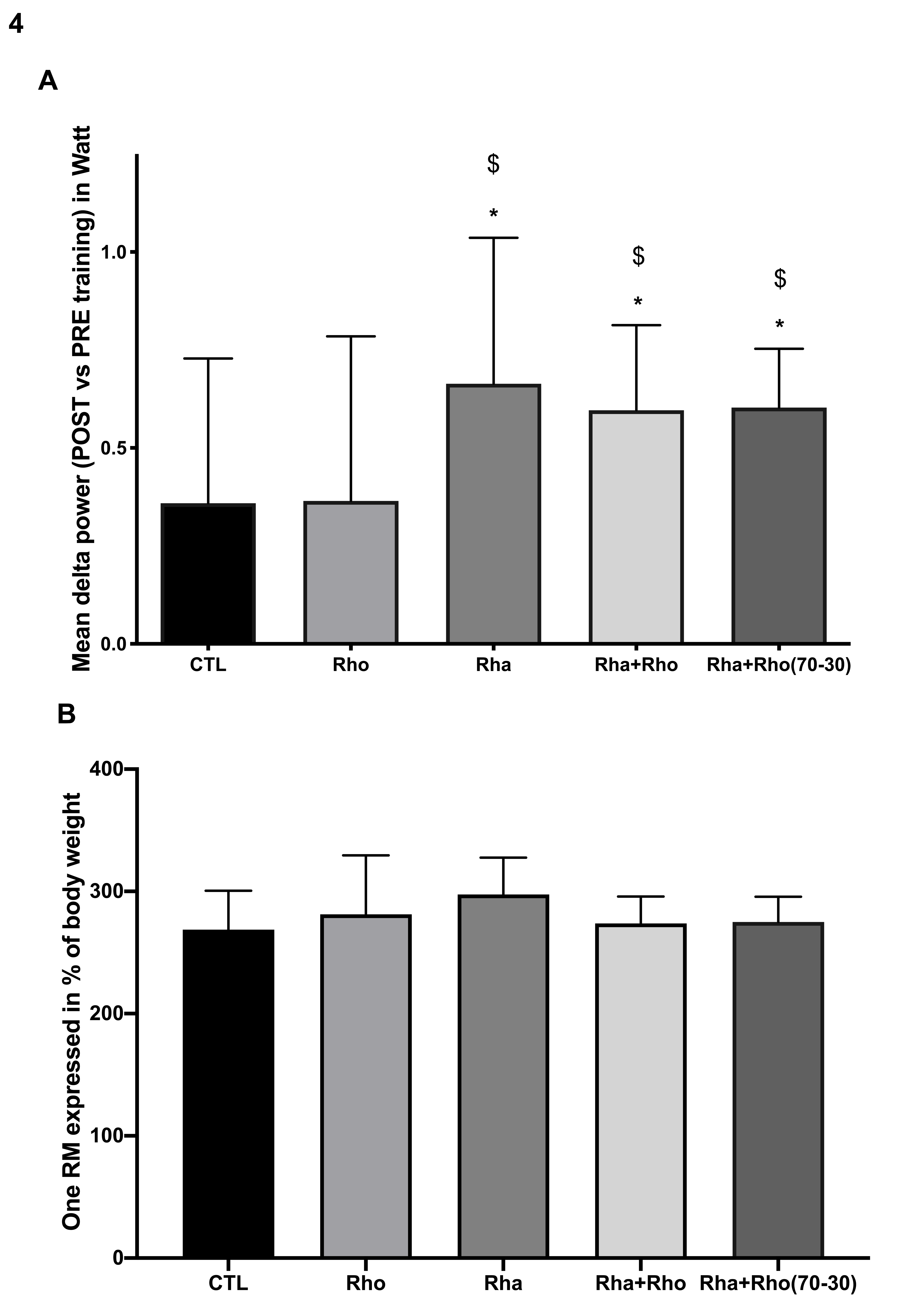

Supplement: Supplementary file 4 — Additional file 4: Figure 4. Effects of chronic Rhaponticum and Rhodiola extract treatments (at the dose of 175 mg Rha (70%) + Rho (30%)) associated with exercise on physical performance. The graphs show the mean delta power between pre-training and post-training in each treatment group (4.A), and for the 1-RM test (4.B). *: p < 0.05 compared to the CTL group. $: p < 0.05 compared to the Rho group. (TIFF 1135 kb) [file 12970_2020_390_MOESM4_ESM.tiff]

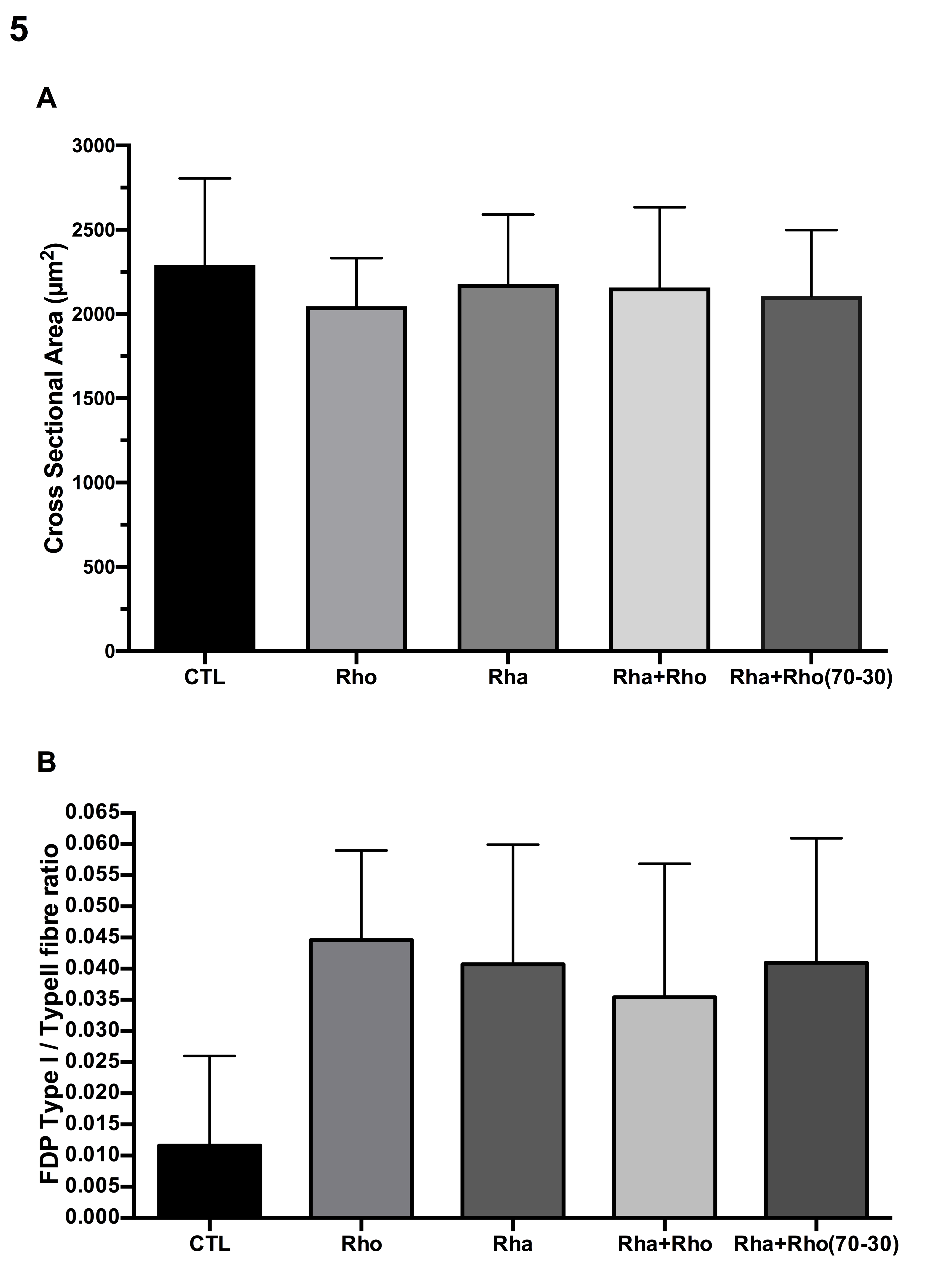

Supplement: Supplementary file 5 — Additional file 5: Figure 5. Effects of chronic Rhaponticum and Rhodiola extract treatments (at the dose of 175 mg Rha (70%) + Rho (30%)) associated with exercise on muscle fibers (FDP muscle). The mean cross-sectional area of FDP muscle is presented in Fig. (5.A). The ratio of type I/type II fibers was evaluated (5.B). [file 12970_2020_390_MOESM5_ESM.tiff]

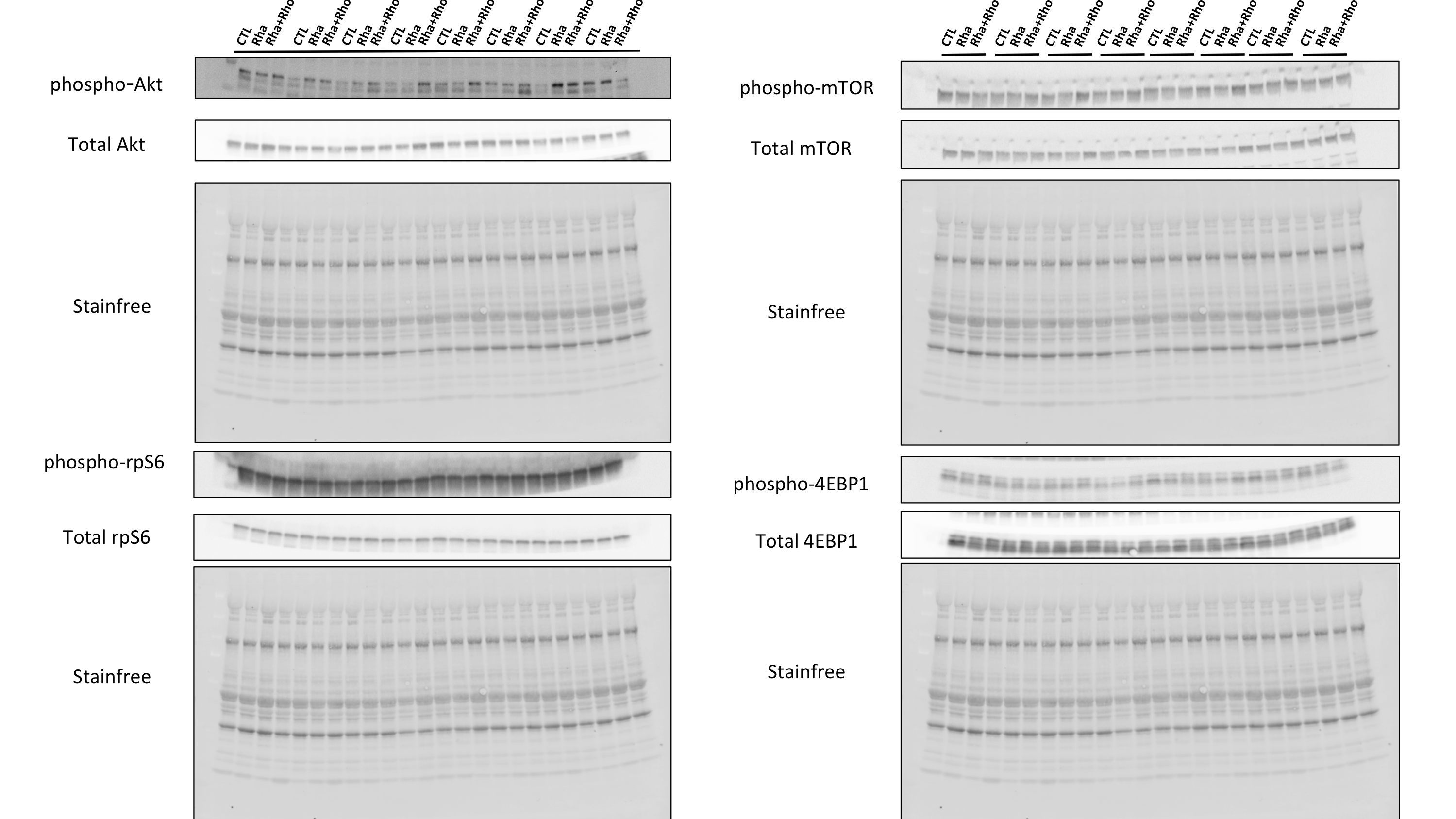

Supplement: Supplementary file 6 — Additional file 6: Figure 6. Illustrations of Western Blot quantitative analysis of p-Akt/total Akt, p-mTOR/total mTOR, p-rpS6/total rpS6 and p-4EBP-1/total 4EBP-1 in total protein extracts of FDP muscle, with Stainfree as internal control. [file 12970_2020_390_MOESM6_ESM.png]

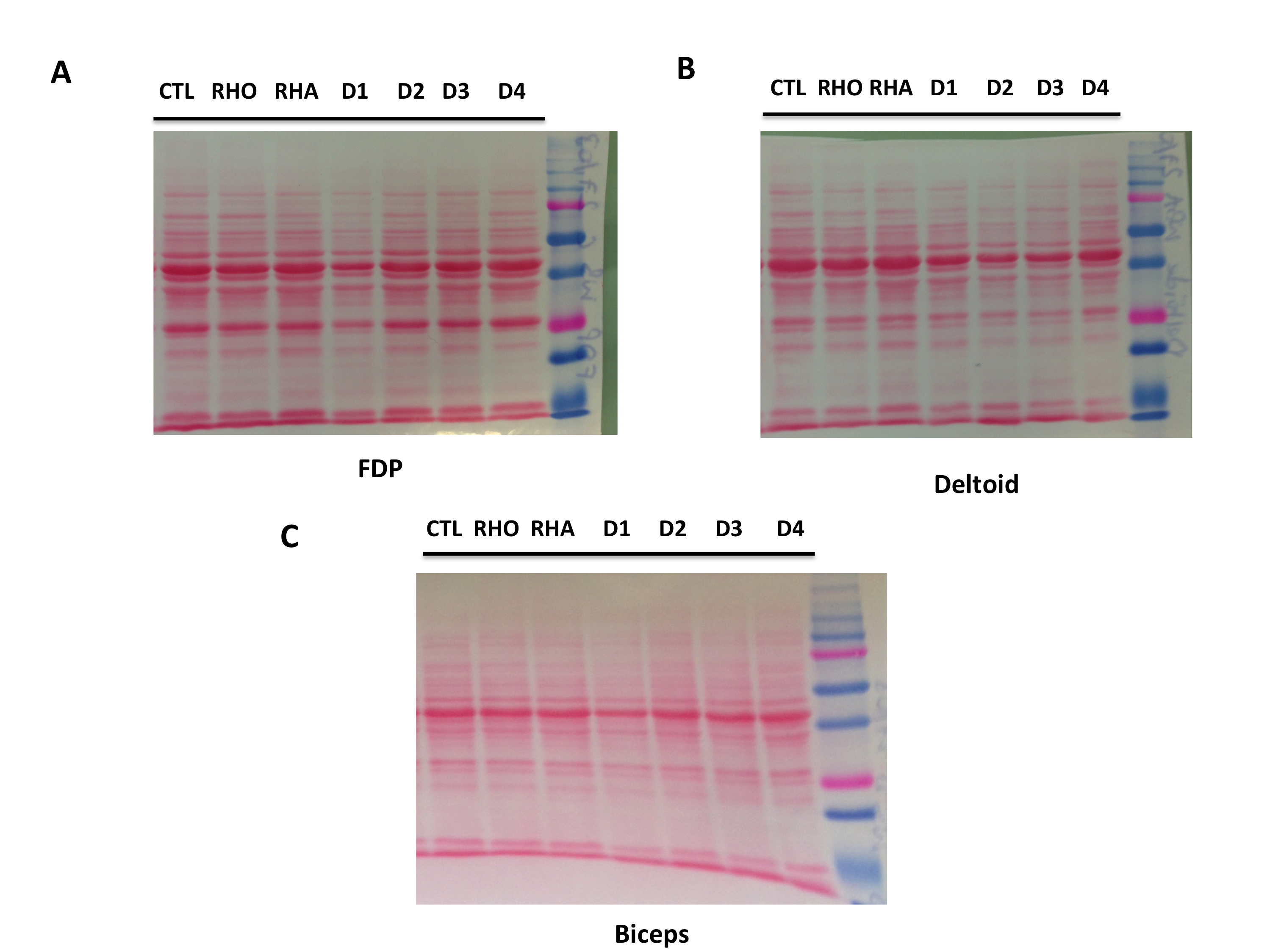

Supplement: Supplementary file 7 — Additional file 7: Figure 7. Illustrations of Ponceau as internal control for analysis of puromycin incorporation in total muscle protein extracts of FDP (A), deltoid (B) and biceps muscles (C). [file 12970_2020_390_MOESM7_ESM.png]
