# Supplementary material for: Acute and chronic effects of Rhaponticum carthamoides and Rhodiola rosea extracts supplementation coupled to resistance exercise on muscle protein synthesis and mechanical power in rats
Source: J Int Soc Sports Nutr. 2020 Nov 16;17:58. doi: 10.1186/s12970-020-00390-5 (PMC7670727; doi:10.1186/s12970-020-00390-5)
Supplement: Supplementary file 8 — Additional file 8: Additional methods. [file 12970_2020_390_MOESM8_ESM.docx]

**ADDITIONAL METHODS:**

Chronic Study Design

*Animals*

In order to a subsequent transfer to the clinic, requiring the product’s cost reduction especially for Rhodiola plant extracts, we designed a new group : eleven-week-old Wistar Han rats (n=10) were fed 30 g/day of the specialized low-protein food A04, previously designed and obtained from SAFE (Augy, France), and water was provided ad libitum. On day 1 of the experiment, the rats were 12 weeks old (334.4 ± 10 g) and were considered adults.

We designed a new dose to test chronically : 175 mg ; *Rha* (70%) + Rho (30%). The rationale for designing the 70%/30% dose was derived from the results of the acute study, but we are aware that our acute study does not provide information regarding this group. We designed a mix dose of 175 mg *Rha* (70%) + Rho (30%) according to the following reasoning : *Rhaponticum* is supposed to drive the anabolic effect upon protein synthesis and *Rhodiola* seems to have a synergistic effect when they are associated, even with low dose (FDP muscle, HED = 50 mg). Based on this observation, we designed a combination to maintain the MPS stimulatory effect keeping the minimum doses of each compound, aiming at reducing the cost of products, in order to a subsequent transfer to the clinic. Based on the results indicating that lowest 50%/50% dose had a significant effect on protein synthesis on most of the muscles studied (HED = 250 mg) (Figure 1) we designed a supplementation where the *Rhaponticum* dose of this combination was preserved (i.e. HED = 125 mg), but the *Rhodiola* dose was decreased to the lowest level that had a synergistic effect (FDP muscle, HED = 50 mg). We used a total dose of 175 mg (Rha = 125 mg + Rho = 50 mg) with a *Rha+Rho* ratio of 70%/30%. Thus, we designed a mix dose of 175 mg *Rha* (70%) + Rho (30%) that was administered to animals that received also resistance training for 4 weeks. Thus, we designed a mix dose of 175 mg *Rha* (70%) + Rho (30%) that was administered to animals that received also resistance training for 4 weeks. Rats received treatment as shown in Table 1.

**Table 1:** Human equivalent doses (HEDs) used to feed the different groups of animals in the chronic study

| **Conversion of the human dose to rat dose** | **Daily rat dose (mg/kg BW)** | **HED (mg/kg BW)*** | **HED (mg/day)** |
| --- | --- | --- | --- |
| *Rhaponticum/Rhodiola* 70-30 group (*Rha+Rho*) | 15.22 | 2.92 | 175 |

* Formula from FDA, 2005; Human equivalent dose (HED; mg/kg) = animal dose in mg/kg x (animal weight in kg/human weight in kg)^0.33^. BW: body weight

*Protein synthesis markers measurement*

Protein synthesis markers expression was assessed by immunoblotting on 4-20% acrylamide gels. Protein samples from FDP muscle (50 µg) were denatured, separated by 10% SDS-PAGE, and transferred onto nitrocellulose membranes. Membranes were incubated with primary antibodies against phospho-Ak (Ser 473), Akt , phospho-mTOR (Ser 2448), mTOR, phospho-rpS6 (ser 240/244), rpS6, phospho-4EBP-1 (ser 65), 4EBP-1 (Cell Signaling Technology, Danvers,MA, USA) overnight at 4°C, and the membranes were subsequently incubated with appropriate secondary antibody conjugated to peroxidase (anti-mouse or anti-rabbit IgG ; ECL from GE Healthcare UK Limited, Amersham, UK) The optical density of the entire sample lane was assessed and normalized with Ponceau S total protein staining.
